# Lung Function Measurements in Rodents in Safety Pharmacology Studies

**DOI:** 10.3389/fphar.2012.00156

**Published:** 2012-08-28

**Authors:** Heinz Gerd Hoymann

**Affiliations:** ^1^Fraunhofer Institute for Toxicology and Experimental MedicineHannover, Germany

**Keywords:** experimental animal models, irritant potential, juvenile models, lung function test, mouse, pharmacology, rat, safety pharmacology

## Abstract

The ICH guideline S7A requires safety pharmacology tests including measurements of pulmonary function. In the first step – as part of the “core battery” – lung function tests in conscious animals are requested. If potential adverse effects raise concern for human safety, these should be explored in a second step as a “follow-up study”. For these two stages of safety pharmacology testing, both non-invasive and invasive techniques are needed which should be as precise and reliable as possible. A short overview of typical *in vivo* measurement techniques is given, their advantages and disadvantages are discussed and out of these the non-invasive head-out body plethysmography and the invasive but repeatable body plethysmography in orotracheally intubated rodents are presented in detail. For validation purposes the changes in the respective parameters such as tidal midexpiratory flow (EF_50_) or lung resistance have been recorded in the same animals in typical bronchoconstriction models and compared. In addition, the technique of head-out body plethysmography has been shown to be useful to measure lung function in juvenile rats starting from day two of age. This allows safety pharmacology testing and toxicological studies in juvenile animals as a model for the young developing organism as requested by the regulatory authorities (e.g., EMEA Guideline 1/2008). It is concluded that both invasive and non-invasive pulmonary function tests are capable of detecting effects and alterations on the respiratory system with different selectivity and area of operation. The use of both techniques in a large number of studies in mice and rats in the last years have demonstrated that they provide useful and reliable information on pulmonary mechanics in safety pharmacology and toxicology testing, in investigations of respiratory disorders, and in pharmacological efficacy studies.

## Introduction

Safety pharmacology studies are necessary for the development of drugs and for protection of clinical trial participants and patients from potential adverse effects. The ICH guideline S7A recommends safety pharmacology tests including measurements of pulmonary function. There are no differences in the guidelines of the European Union, the USA and Japan since ICH S7A has been adopted by the EMA, the FDA, and the MHLW. Their objective is to identify potential adverse or undesirable effects of a compound in relation to dosage within the compounds therapeutic range and above. Those safety pharmacology studies on the respiratory system are typically small studies, mostly independent from toxicological studies, with single treatment or inhalation exposure conducted in accordance with GLP guidelines for regulatory submission. Usually these studies are performed in rodents (mostly in rats, rarely in mice).

The principles governing ventilation, air flow, lung volume, and gas exchange are common among most if not all mammals (Costa and Tepper, 1988). Inhalation toxicological studies and studies using specific experimentally induced lung diseases in animals have shown that functional responses of man and animals to different types of lung injury are similar (Mauderly, 1988, 1995). Examination of pulmonary function is a non-destructive procedure of assessing the functional consequences of alterations of lung structure or (temporary) changes in the tonus of airway smooth muscle cells, providing information on the presence, the type, and the extent of alteration (Mauderly, 1989). Existing methods for measuring respiratory function *in vivo* include non-invasive and invasive technologies.

Changes in respiratory function can result either from alterations in the pumping apparatus including nervous and muscular components that controls the pattern of pulmonary ventilation or from changes in the mechanical properties of the gas exchange unit consisting of the lung with its associated airways, alveoli, and interstitial tissue (Murphy, 2002). Defects in pumping apparatus and reflex-related alterations can change the breathing pattern and are tested non-invasively in a conscious animal model. Defects in mechanical properties of the lung can result in obstructive or restrictive disorders which often can also be detected by non-invasive lung function parameters but can be better evaluated by invasive lung function tests and pulmonary maneuvers in anesthetized animals due to their higher sensitivity and specificity.

## Lung Function Measurement Techniques for Rodents – An Overview

Lung function is a relevant endpoint in *pharmacological studies* (e.g., in models for asthma, COPD, or infection), in *safety pharmacological studies* performed according to the ICH guideline S7A (core battery, part lung, and follow-up), and finally in *toxicological studies*, particularly if the airways are in the focus of interest (e.g., tests on allergenic or irritant potential and functional tests on obstructive or restrictive lung alterations or diffusion disorders). The safety pharmacological and toxicological studies have to be performed in compliance with the GLP Principles.

The existing methods to measure pulmonary function in rodents *in vivo* are divided in invasive and non-invasive approaches which all have their advantages and disadvantages (for short overview, see Table 1). However, such experiments present particular technical challenges, and each method lie somewhere along a continuum on which non-invasiveness must be traded off against experimental control and measurement precision (Bates and Irvin, 2003). As an extreme of non-invasiveness unrestrained plethysmography (Penh) in conscious mice or rats is highly convenient but provides respiratory measures that are so tenuously linked to respiratory mechanics that they were seriously questioned recently by several authors (Lundblad et al., 2002; Mitzner and Tankersley, 2003; Adler et al., 2004; Bates et al., 2004), discussed in detail in Section “Advantages and Disadvantages of Invasive and Non-Invasive Techniques”. At the other extreme, the measurement of input impedance in anesthetized, paralyzed, tracheostomized mice is precise and specific but requires that an animal be studied under conditions far from natural (Bates and Irvin, 2003).

**Table 1:** **Short overview of *in vivo* methods used to measure lung function in rodents**.

Method	Principle
**Non-invasive** techniques – with **unrestrained** animals:	**“Controlling nothing”**
• Barometric plethysmography (“Penh system”)	Hamelmann et al. (1997)
**Non-invasive** techniques – with **restrained** animals:	**“Controlling something”**
• Head-out plethysmography	Vijayaraghavan et al. (1993) Glaab et al. (2002)
• Double chamber plethysmography	*Guinea pig*: Pennock (1979) *mouse*: Flandre et al. (2003)
• Forced oscillation technique	Hessel et al. (1995)
**Invasive** plethysmography in spontaneously breathing, mostly anesthetized animals:	**“Controlling more”**
• in orotracheally intubated animals **(repetitive)**	Rat: Likens and Mauderly (1982) Mouse: Brown et al. (1999) Glaab et al. (2004)
• pulmonary maneuvers in intubated rats **(repetitive)**	Likens and Mauderly (1982)
• after surgical implantation of pleural pressure sensor chronic resistance recording in conscious rats **(repetitive)**	Murphy et al. (1998)
• in tracheostomized, intubated animals **(non-repetitive)**	Palecek et al. (1967)
**Invasive** plethysmography in ventilated, anesthetized animals:	**“Controlling everything”**
• Forced oscillation technique **(non-repetitive)**	*rat*: Jackson and Watson (1982) *mouse*: Schuessler and Bates (1995)

*Reprinted with permission from Hoymann (2007), modified*.

In the following two well-established methods – an invasive and a non-invasive technique – are presented which we use in our labs at the Fraunhofer ITEM to record lung function repetitively in rats and mice *in vivo*. In safety pharmacology studies of the stage one (core battery) as well as in tests on irritant potential the non-invasive head-out plethysmography technique is used. In safety pharmacology stage two – the follow-up studies – or in some toxicological studies which both need the most sensitive endpoints the invasive technique in intubated rodents is used.

### Non-invasive head-out plethysmography in conscious rodents for safety pharmacology core battery studies

The ICH guideline S7A requires the assessment of effects on the respiratory system as one of the three “vital organ systems that should be studied in the core battery”. In this first stage of the safety pharmacology package mostly non-invasive techniques are used in conscious rodents which avoid the need of anesthesia. Yves Alarie and coworkers have shown already in 1993–1994 that this technique is highly useful to assess effects on breathing pattern and to detect sensory irritation, pulmonary irritation, and airflow limitation (Vijayaraghavan et al., 1993, 1994). Subsequently, a lot of studies have been performed using head-out plethysmography for validation purposes and to test on adverse effects of chemicals and drugs, amongst others with inhalation exposure to allergens or bronchoconstricting agents (Neuhaus-Steinmetz et al., 2000; Glaab et al., 2001, 2002, 2005; Hoymann et al., 2009; Legaspi et al., 2010; Nirogi et al., 2012). Today, the head-out body plethysmography is a well-established and widely accepted technique which has been proven as a reliable method to measure pulmonary function (for reviews, see Murphy, 2002; Hoymann, 2006, 2007; Renninger, 2006; Glaab et al., 2007).

**Figure 1:**
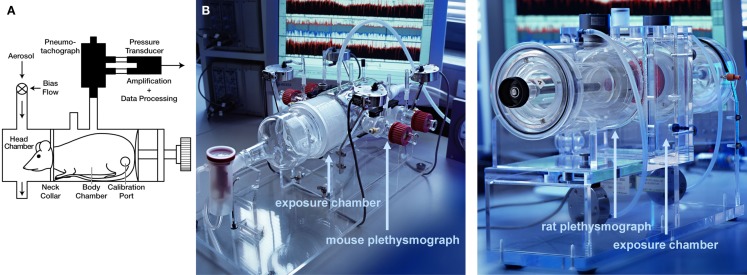
**Head-out plethysmography system**. **(A)** Schematic drawing of rodent head-out plethysmograph [reprinted with permission from (Glaab et al., 2001), modified] made of glass or Plexiglas^®^. The plethysmographs are attached to a head exposure chamber. Respiratory flow is measured by means of pneumotachograph tube connected to a pressure transducer (see text for details). **(B)** Photos of head-out plethysmography systems for four mice (left) or four rats (right; only one of two systems shown).

Prior to the measurements, the animals are trained for 5 days in increasing time periods to get accustomed to the plethysmograph. For lung function measurements for the core battery, the animals are placed in body plethysmographs while the head of each animal protrudes through a neck collar of a dental latex dam into a head exposure chamber (see Figure 1). This chamber consists of a 5.9 L Plexiglas^®^ cylinder in the rat system which is ventilated with a continuous bias flow of ca. 1 L/min for two rats for lung function measurements using a source of compressed air, but for aerosol administration to produce well-defined stable exposure conditions often a much higher bias flow is necessary (e.g., a bias flow of ca. 10–20 L/min when using a jet-driven aerosol generator). Two systems are combined to measure lung function in four rats simultaneously. For mice the head chamber consists of a 2.5 L glass cylinder which is ventilated with a bias flow of ca. 0.5 L/min for four mice.

Monitoring of pulmonary function is started when animals and individual measurements settled down to a stable level (“steady state,” after about 4–5 min in rats). The respiratory flow is measured as the flow through a calibrated pneumotachograph connected with the plethysmograph and caused by the thoracic movements of the animal. The flow is measured by using a differential pressure transducer connected with the pneumotachograph. From the amplified flow signals several parameters are obtained: the tidal volume (*V*_T_) of the spontaneously breathing animal in mL, its respiratory rate (*f*, in min^−1^), the respiratory minute volume (MV, mL), the tidal midexpiratory flow (EF_50_, mL/s, see below), and the time of in- and expiration (TI, TE; time taken to inspire/expire, ms) are calculated for each breath with a commercial software (HEM, Notocord, France). In addition, two parameters can be evaluated which can indicate irritation effects: the time of brake (TB) quantifies an elongation of the period from the end of the inspiration until the start of the expiration and the time of pause (TP) quantifies an elongation of the period from the end of the expiration until the start of the new inspiration (in ms).

**Figure 2:**
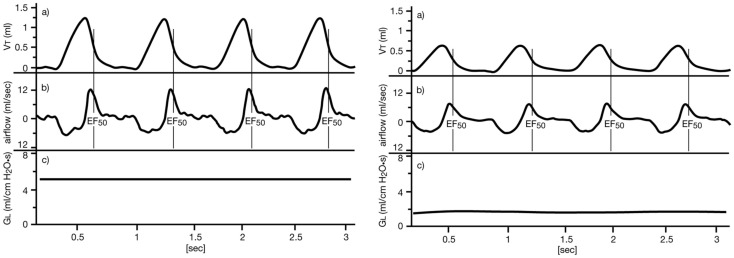
**Definition of midexpiratory flow (EF_50_)**. Left: normal breathing pattern of an anesthetized, orotracheally intubated, spontaneously breathing BN rat. Right: breathing pattern during bronchoconstriction due to inhalation of ca. 15 μg ACh aerosol, illustrating the simultaneous decreases in EF_50_ and *G*_L_. Upper tracing (*V*_T_): tidal volume (*V*_T_) obtained from the integrated airflow signal over time, a vertical line indicates the value of EF_50_ at midexpiratory *V*_T_. Middle tracing: corresponding airflow signal from the pneumotachograph during expiration (above zero) and inspiration (below zero). Lower tracing (*G*_L_): corresponding lung conductance *G*_L_ = 1/R_L_ [reprinted with permission from Glaab et al. (2002)].

If an airflow limitation is present, e.g., caused by bronchoconstriction, edema, or accumulation of mucus, the main changes in the tidal flow signal occur during the midexpiratory phase: EF_50_ (mL/s) is defined as the tidal flow at the midpoint (50%) of expiratory *V*_T_ (see Figure 2), and is used as a measure of bronchoconstriction/-obstruction (Glaab et al., 2001, 2002, 2005; Hoymann, 2007). We and others have described the utility of EF_50_ as a physiologically meaningful, non-invasive parameter of bronchoconstriction for rats and mice (Vijayaraghavan et al., 1994; Neuhaus-Steinmetz et al., 2000; Finotto et al., 2001; Glaab et al., 2001, 2002, 2005; Hantos and Brusasco, 2002; Hoymann, 2007; Nirogi et al., 2012). The degree of bronchoconstriction in response to inhalation challenge was determined from minimum values of EF_50_ and was expressed as percent changes from corresponding baseline values.

**Figure 3:**
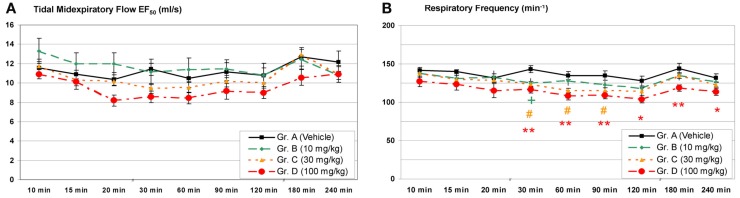
**Head-out plethysmography in a safety pharmacology core battery study: (A)** Tidal midexpiratory flow (EF_50_) and **(B)** respiratory frequency measured after a single intragastric treatment with a pharmacologically active test compound [mean ± SEM; +/#/* = low/medium/high dose *p* < 0.05, ^**^ = high dose *p* < 0.01 vs. control group; reprinted with permission from Hoymann (2007)].

The group size recommended for safety pharmacology studies is *n* = 8 for standard studies. As an example the time course of typical lung function parameters from a core battery study is given in Figure 3: Male Brown Norway rats (*n* = 8/group) have been treated with a pharmaceutical test compound intragastrically in three dose groups and then lung function was monitored using head-out plethysmography. In the week prior to the measurements, the animals were trained for 5 days in increasing time periods to get accustomed to the plethysmograph (“tube training”). Typical parameters of pulmonary function have been measured in this study: the tidal midexpiratory flow (EF_50_), the tidal volume (*V*_T_), the respiratory frequency (*f*), and the time of inspiration (TI) and expiration (TE). From these parameters EF_50_ and f are shown in Figure 3. No significant effect on EF_50_ was observed during 4 h after treatment. In contrast, a dose-dependent decrease of the frequency was found – in this case probably due to a central-nervous effect of this test compound.

#### Experimental comparison of EF_50_ with invasive lung function parameters

**Figure 4:**
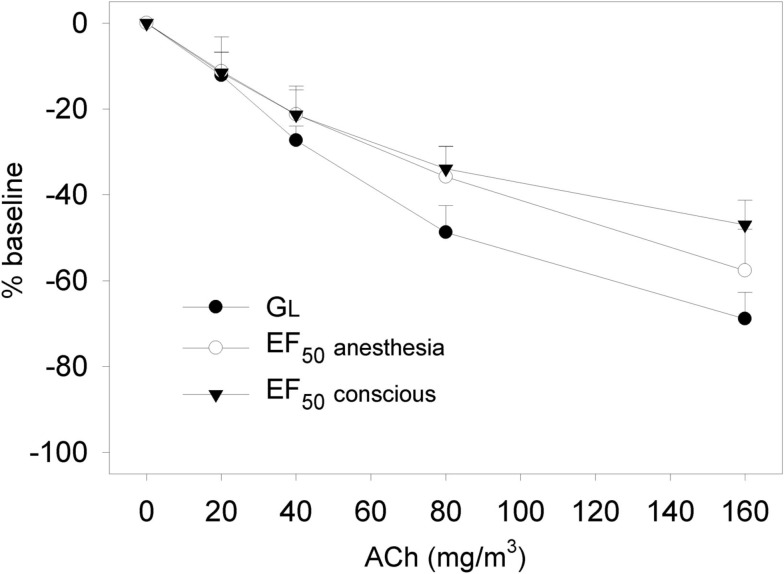
**Dose-response relationship to aerosolized acetylcholine chloride (ACh; 20–160 mg/m^3^) in naive Brown Norway rats**. Non-invasive determination of the decline in EF_50_ to ACh was followed by invasive recording of simultaneously measured decreases in EF_50_ and *G*_L_ (*G*_L_ = 1/R_L_) to ACh exposure in the same animals 24 h later. EF_50_ and *G*_L_ were allowed to return to baseline before each subsequent challenge. Results are means ± SD (*n* = 8 rats) of percent changes to corresponding baseline values, which were taken as 0%. No significant differences in dose-related changes were observed between non-invasively and invasively measured EF_50_. [reprinted with permission from Glaab et al. (2002)].

Several studies have been performed to compare the parameter EF_50_ with invasively measured gold standard parameters for validation purposes. In particular, lung function was measured invasively and non-invasively in Brown Norway rats (see Figure 4) and BALB/c mice as standard strains for safety pharmacological and pharmacological studies. These experiments were performed with inhalation exposure to allergens such as ovalbumin and *Aspergillus fumigatus* extract as well as to the bronchoconstrictors methacholine and acetylcholine (Glaab et al., 2001, 2002, 2005, 2006). The results of these studies showed a good correlation of EF_50_ with the classical invasive measurements of lung resistance and dynamic compliance with a somewhat lower sensitivity and greater variability of EF_50_. The measurement of EF_50_ is particularly appropriate for quick and repeatable screening of respiratory function in large numbers of rodents or if non-invasive measurement in mice and rats without use of anesthesia is required. These data support the use in safety pharmacology core battery part respiratory system.

#### Tests on irritant effects (the Alarie test)

A standard bioassay for testing of airborne substances as, e.g., industrial chemicals on potential irritant effects is the well-established Alarie test. In the case that a drug is tested on irritant potential this test can be a special form of a safety pharmacological study or an extension of it. The Alarie test uses the same equipment and technique as described above (see Non-Invasive Head-out Plethysmography in Conscious Rodents for Safety Pharmacology Core Battery Studies): the head-out plethysmography in conscious mice. The test was standardized by the American Society for Testing and Materials (ASTM, 1984). A decade later the extended computerized version of the Alarie test was created to determine effects on three levels of the respiratory system (Vijayaraghavan et al., 1993, 1994): the upper respiratory tract (i.e., sensory irritation), the conducting airways (airflow limitation) and the alveolar level (pulmonary irritation). The Alarie test is very well validated: An excellent correlation was found between RD50 values (see below) of 89 substances and Threshold Limit Values (TLV) of human exposure representing possibly the largest data base in toxicology (Schaper, 1993).

If a substance stimulates the trigeminal nerve endings in the upper respiratory tract of mice, which in humans may result in a burning and painful sensation, it causes a reflexively induced decrease in the respiratory rate (*f*; Vijayaraghavan et al., 1993). This decrease is caused by an elongation of the period from the end of the inspiration until the start of the expiration, termed “TB”. Therefore, the sensory irritation can be detected by a decrease in *f* using the concentration at which a respiratory decrease of 50% is reached (“RD50”) but a more specific detection is possible by directly using the TB in the modern form of the Alarie test. Additionally, airflow limitation can be detected by using the EF_50_ as already described above.

Stimulation of vagal nerves at the alveolar level may result in two types of respiratory effects. One effect – usually found at lower concentrations and beginning irritation effect – is rapid, shallow breathing which increases f and reduces *V*_T_. At higher concentration and effect level an increase in the TP is observed which is the time period from the end of the expiration to the initiation of the following inspiration. Therefore, the latter form of pulmonary irritation causes a decrease in *f* and can be quantified by it and the term RD50, but TP is the more specific of the two parameters (Vijayaraghavan et al., 1994).

### Invasive lung function measurement in orotracheally intubated rodents for safety pharmacology follow-up studies

The ICH guideline S7A requires extended measurements of pulmonary function as follow-up study if adverse effects may be suspected based on the pharmacological properties of the test compound or if the results of a conducted core battery study give rise to concerns. Especially, if the core battery indicates, e.g., flow limitation by a decrease in EF_50_ or a rapid shallow breathing pattern, the mechanical properties of the lung can be further evaluated functionally by invasive lung function tests or pulmonary maneuvers in anesthetized animals using their higher sensitivity and specificity. For the measurement of lung resistance and compliance a pressure-sensitive catheter has to be inserted into the pleural cavity or the esophagus for the measurement of pleural, airway, or transpulmonary pressure. Therefore, the animals are generally anesthetized.

**Figure 5:**
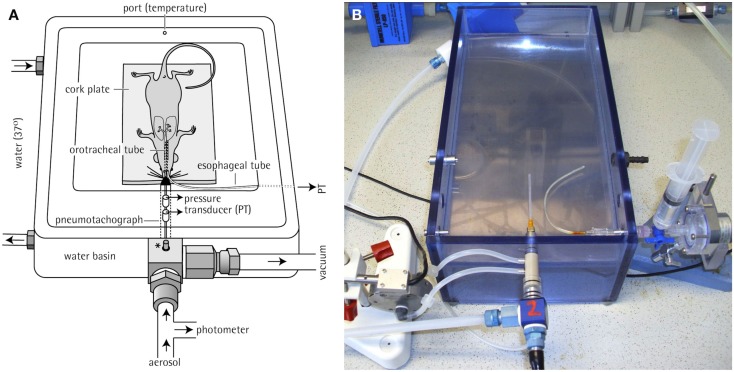
**Invasive body plethysmography system**. **(A)** Diagram of a plethysmograph used for pulmonary function testing of anesthetized, orotracheally intubated rodents [shown is a unit for a mouse, reprinted with permission from Glaab et al. (2004)]. A thermostat-controlled water basin (37°C) is built in the plethysmograph chamber to avoid decrease in body temperature. *: adaptor connected to a pressure control unit that maintains constant pressure conditions during measurements. **(B)** Photo of a plethysmograph unit for a rat. For the calculation of R_L_, P_TP_ was recorded via an esophageal tube and tidal flow was determined by a pneumotachograph tube attached directly to the orotracheal tube.

Invasive measurements of pulmonary function in rodents with the option of simultaneous aerosol inhalation are facilitated by careful orotracheal intubation in spontaneously breathing animals such as the rat (Hohlfeld et al., 1997; Hoymann and Heinrich, 1998; Hoymann, 2007) or the mouse (Glaab et al., 2004, 2005; Hoymann, 2006, 2007) which yield the feasibility of repetitive experiments in the same individuals. Briefly, the animals are anesthetized by injection and/or inhalation of volatile anesthetics: e.g., for rats with a combination of 13 mg/kg pentobarbital sodium i.p. +0.8% isoflurane by inhalation or with 1.3–1.7% isoflurane alone, and for mice with a combination of 35 mg/kg pentobarbital sodium i.p. +0.8–1% isoflurane or with a combination of 23 mg/kg Etomidate i.p. +0.05 mg/kg Fentanyl i.p. When an appropriate depth of anesthesia is achieved, the rodents are intubated carefully orotracheally under visual control by transillumination of the neck. For rats, the tracheal cannula is prepared from a Cathlon IV 14G intravenous catheter (ID 1.78 mm, OD 2.1 mm, length reduced to 52 mm). For mice, a tracheal tube made from Teflon^®^ (Abbocath^®^-T 20Gx32, ID 0.80 mm, OD 1.02 mm, length 32 mm) or, alternatively, made from steel is used. After intubation, the spontaneously breathing animal is placed in supine position in a body plethysmograph (see Figure 5). In 2002–2004, we developed a plethysmograph system for invasive lung function measurement with simultaneous inhalation administration in anesthetized mice in cooperation with Hugo Sachs Elektronik/Harvard Apparatus (Glaab et al., 2004). A thermostat-controlled warming pad [mice: water basin (37°C)] built in the plethysmograph chamber ensures a normal body temperature. The animals can breathe air spontaneously out of a tubing system providing the animal with air containing 30–40% oxygen to prevent hypoxia by using a source of compressed air (bias flows of ca. 0.8–1.2 L/min per animal used for two rodents simultaneously).

The orotracheal cannula of each animal is directly attached to a capillary pneumotachograph tube (rats: PTM 378/1.2, mice: PTM T16375; HSE-Harvard) installed in the front part of the chamber. The pneumotachograph tube is connected to a differential pressure transducer to determine tidal respiratory flow. A water-filled PE-90 tubing is inserted into the esophagus to the level of the midthorax and coupled to a pressure transducer to measure transpulmonary pressure (*P*_TP_). By processing these two primary signals lung resistance (*R*_L_) and dynamic compliance (C_dyn_) can be calculated which are known as the gold standard parameters of lung function, especially when assessing bronchoconstricting or -obstructing effects. These parameters are defined as

(1) $$R_{L}=\frac{\Delta P_{\text{TP}}}{\Delta F}\text{ and }C_{\text{dyn}}=\frac{\Delta V}{\Delta P_{\text{TP}}}.$$

By using the isovolumetric method (Amdur and Mead, 1958) or by using an integration method applied to flow, volume and pressure signals (Roy et al., 1974; Glaab et al., 2004) – which we prefer in our labs – R_L_ and C_dyn_ are calculated over a complete respiratory cycle (software: HEM/Notocord). Recording of pulmonary function is started when the measured signals reached a stable level (“steady state”). The recommended group size is comparable to non-invasive technique: for standard safety pharmacology studies *n* = 8 rats or mice.

**Figure 6:**
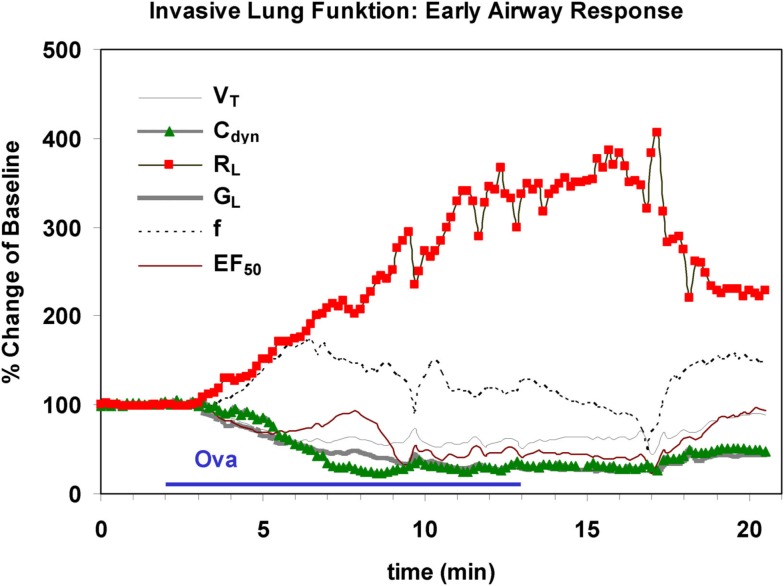
**Characteristic time course of an early airway response in an anesthetized, orotracheally intubated Brown Norway rat during and after inhalational challenge with ovalbumin**. Marked increase in lung resistance (R_L_) is shown and a decrease in dynamic compliance (C_dyn_) is paralleled by decreases in lung conductance (*G*_L_), midexpiratory flow (EF_50_) and tidal volume (*V*_T_). Also a slight increase in respiratory frequency (*f*) was observed. The x-axis represents the experimental time (unit = 1 min).

This plethysmography technique is combined with an appropriate inhalation system for intubated mice or rats which allows simultaneous lung function measurement. Inhalation treatments or provocations via the orotracheal tube are performed using an effective and computer-controlled aerosol generator such as the Bronchy III (constructed in Fraunhofer ITEM) with an effective drying system: solutions of drugs or provocative agents are sprayed into an evaporation chamber warmed to, e.g., 40°C and dried, the solvent is removed and the aerosol re-cooled to 25°C in a diffusion dryer module and is then conducted to the animal (Hoymann, 2006). The exact dose is calculated and controlled by a computerized feedback dose-control system (Fraunhofer ITEM) which has been successful in generating constant dosing in rats and mice (Hoymann and Heinrich, 1998; Glaab et al., 2004, 2005; Hoymann, 2006). Based on the inspiratory aerosol concentrations continuously measured by a gravimetrically calibrated photometer and the respiratory MV (mL/min) the exact inhalation doses are calculated by this system. The software allows to pre-select a dose in μg and an exposure time (Hoymann, 2006). For example: if an inhalation dose of 300 μg and an exposure time of 10 min is preselected, the system controls the aerosol concentration to reach 300 μg in 10 min in each animal independently of the MV of this animal. If the MV rises the concentration is decreased (by increasing a dilution air flow via mass flow controllers) and vice versa. This results in a constant dose and also dose/time relation in each animal. An example is given in Figure 6: a marked increase in R_L_ and decrease in C_dyn_ is shown during and after inhalational exposure of a sensitized rat with the allergen.

### Lung function measurements in juvenile rats

Most drugs intended for use in children have not been formally developed for use in this age group. As juvenile animal studies are requested more and more often by the regulatory authorities during drug development, the need to modify standard experimental procedures to be applied to neonatal and juvenile animals and to provide basic data on respiratory parameters is becoming urgent (European Commission, 2006a,b). The European Medicines Agency (EMA, before 2009: “EMEA”) published a “Guideline on the Need for Non-clinical Testing in Juvenile Animals of Pharmaceuticals for Pediatric Indications” in 2008 (EMEA, 2008): Approval of these medicinal products for pediatric use “requires a special risk/benefit assessment, where the possible effects of the product on the ongoing developmental processes in the age group(s) to be treated are also taken in consideration. This risk/benefit assessment should be based on safety and pharmacokinetic data from non-clinical and clinical studies”.

**Figure 7:**
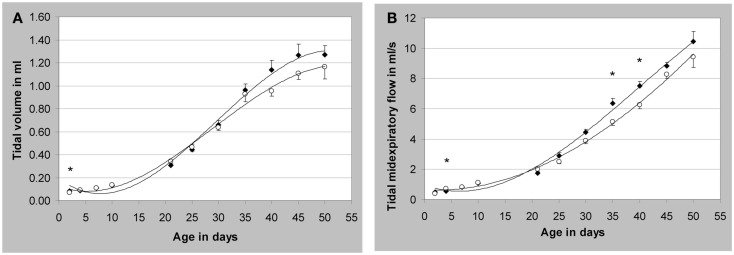
**Development of respiratory parameters in non-anesthetized juvenile Wistar rats**. **(A)** Tidal volume; **(B)** tidal midexpiratory flow; *p < 0.05 male vs. female; mean values ± SEM, measured values of *n* = 8/sex (except males on post-natal day (PND) seven and females on PND 21 and 45: *n* = 7). Closed symbols represent male animals (♦) and open symbols represent female animals (○). [reprinted with permission from Lewin et al. (2010)]; see there for additional data).

Therefore, we have developed a technique based on modified head-out plethysmography (see chapter Non-Invasive Head-out Plethysmography in Conscious Rodents for Safety Pharmacology Core Battery Studies) to measure pulmonary function non-invasively in juvenile rats between post-natal days (PNDs) 2 and 50 (Lewin et al., 2010). Briefly, the measurements in juvenile rats were taken with equipment designed for mice on PND 2, 4, 7, and 10, and with equipment designed for rats on PND 21, 25, 30, 35, 40, 45, and 50. For airflow measurement, a calibrated pneumotachograph (for rats up to PND 10: a capillary tube PTM 378/1.2, for rats > PND 10: a wire mesh pneumotachometer with six layers of wire mesh cloth, HSE-Harvard, March-Hugstetten, Germany) and a differential pressure transducer (Validyne DP 45-14, HSE-Harvard) coupled to an amplifier were attached to each plethysmograph. Typical lung function parameters were recorded for approximately 15 min in four animals simultaneously. Two examples are given in Figure 7. Each time point was represented by two litters of four males and four females each. All pups were weighed before each measurement and observed for possible clinical symptoms after the measurement.

The methods proved to be feasible and did not interfere with normal growth and development of the animals. This technique in juvenile rats therefore permits new insights to support human neonatal risk assessment and therefore this animal model is suitable for regulatory studies.

### Discussion: Advantages and disadvantages of invasive and non-invasive techniques

As has been shown invasive and non-invasive methods for measurement of lung function both have their advantages and disadvantages (see Table 1). The non-invasive head-out body plethysmography in conscious rodents is simple to handle and the breathing pattern is nearly natural since no anesthesia is required (see Table 2). On the other hand there is a certain amount of stress for the animal (but limited by using “tube training”), lung resistance and compliance cannot be obtained since a P_TP_ signal is not available (therefore EF_50_ is used to describe flow limitation). Though Murphy et al. (1998) have introduced a technique with implanting a pressure-sensitive catheter that resides below the serosal layer of the esophagus to enable direct measurement of sub-pleural pressure. This does not appear to be widespread due to the limitations and the surgical procedure necessary. Ewart et al. (2010) tried to validate this telemetry technique but they found that the pressure signal in the telemetered rats was extremely variable and concluded that assessment of airway resistance is best confined to the anesthetized rat. In addition, the inhalation exposure when using conscious rodents includes nasal and gastro-intestinal uptake which is not desired in special cases.

**Table 2:** **Advantages and disadvantages of a non-invasive and an invasive plethysmographic method**.

**Non-invasive head-out body plethysmography**
Is performed in spontaneously breathing conscious rats or mice
Includes measurement during inhalation exposure
[+]Nearly natural breathing pattern, simple handling, higher throughput
[−]Stress, volume and flow derived parameters but no resistance, and compliance measurable, inhalation exposure includes nasal and gastro-intestinal uptake
**Invasive body plethysmography (orotracheally intubated animals)**
Is performed in anesthetized but spontaneous breathing rats or mice
Includes measurement during inhalation exposure with optimal dose-control
[+]No stress, gold standard parameters resistance, and compliance available, inhalation exposure is focused to the lungs
[−]Anesthesia, decreased breathing frequency, not very simple handling

*Reprinted with permission from Hoymann (2007), modified*.

In comparison, the invasive body plethysmography in orotracheally intubated but spontaneously breathing rodents requires anesthesia of the animals, more training of the technicians, and more time to conduct the measurements. Anesthesia has a depressant effect on respiration which decreases the breathing frequency and changes the breathing pattern. On the other hand, the parameters airway resistance and dynamic compliance are available which are known to be the “gold standard” for detection and quantification of bronchoconstriction and – obstruction (Glaab et al., 2005; Hoymann, 2007; Ewart et al., 2010). Inhalation exposure in the orotracheally intubated animal is focused to the lungs since nose and skin exposure as well as oral intake are excluded.

Therefore, advantages and disadvantages have to be compared in relation to the aim of the study to decide whether invasive or non-invasive lung function should be chosen. Non-invasive measurement of lung function in conscious animals preferentially by head-out plethysmography is recommended for the core battery of safety pharmacology testing or is used if natural breathing pattern is important (with parameters such as midexpiratory flow EF_50_, time of expiration, TB, and TP) or if “high throughput” measurements and a simple technique are important. Invasive lung function testing in orotracheally intubated animals is preferred if the most sensitive and specific parameters such as lung resistance and dynamic compliance are required and therefore are recommended for follow-up studies of safety pharmacology testing, or if controlled inhalation administration into the lungs without other pathways (nose, stomach, skin) or with high deposition doses of drugs or agents are required or if pulmonary maneuvers are desired.

#### Reliable non-invasive measurement – comparison of EF_50_ with Penh

Some years ago the application of the empiric variable enhanced pause (Penh) had gained widespread popularity – also in safety pharmacology core battery studies – due to its simple and convenient handling. But then the criticism arose on the side of the experts in the field and the reviewers of the scientific journals. Unrestrained plethysmography (Penh) provides respiratory measures that are so tenuously linked to respiratory mechanics that they were seriously questioned recently by several authors (Lundblad et al., 2002; Mitzner and Tankersley, 2003; Adler et al., 2004; Bates et al., 2004). Penh is an empiric variable which has been shown to be primarily related to ventilatory timing and unrelated to airway resistance (Mitzner and Tankersley, 1998, 2003; Lundblad et al., 2002). Several studies have shown that changes in Penh and respiratory resistance sometimes do not correlate (Petak et al., 2001; DeLorme and Moss, 2002; Flandre et al., 2003; Adler et al., 2004; Pauluhn, 2004) which leads to misinterpretation. Therefore, a correspondence written by 22 leading experts in the field (Bates et al., 2004) to the editors of the AJRCMB emphasized the danger of the increasing uncritical use of Penh, with potentially misleading assessment of pulmonary function in animal models of lung disease. In addition, many authors have claimed not to use this simple technique and the parameter Penh to reflect airway function without an independent assessment of airway resistance (Drazen et al., 1999; Hantos and Brusasco, 2002; Bates and Irvin, 2003; Kips et al., 2003).

**Figure 8:**
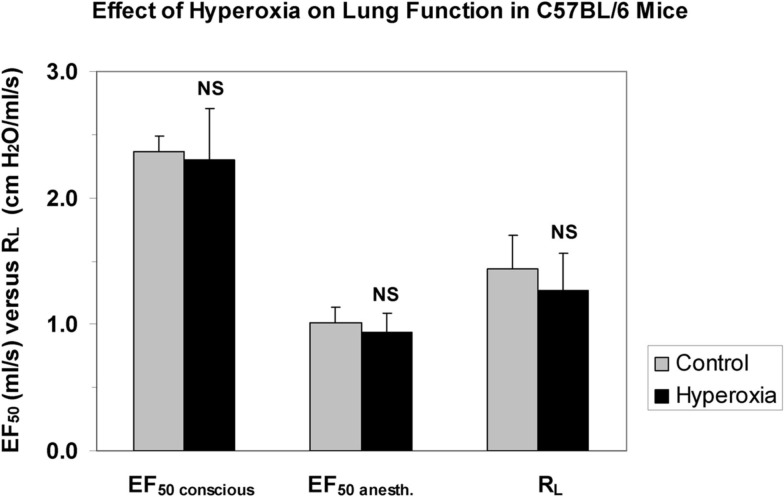
**No impact of hyperoxia shows good correlation of invasively and non-invasively measured respiratory parameters in mice (Glaab et al., 2005)**. Lung function was measured in head-out plethysmographs and subsequently the mice were anesthetized, orotracheally intubated, and lung function was measured invasively. Values are means ± SD of 8 C57BL/6 mice per group exposed to 100% oxygen for 48 h or clean air (control). NS, not significant (vs. control group). EF_50 conscious_: tidal midexpiratory flow of conscious mice, EF_50 anesth._: tidal midexpiratory flow of anesthetized mice, R_L_: lung resistance.

In order to compare and contrast differences in methods, a short comparison of the non-invasively measured EF_50_ (head-out plethysmography) with the non-invasively measured Penh (whole body plethysmography) is given in Table 3. In addition, to support the argument that non-invasive EF_50_ measurement is valid in contrast to Penh we conducted an experiment to examine whether EF_50_, unlike Penh, parallels the actual changes in pulmonary mechanics in response to hyperoxia in C57BL/6 mice. Whereas Petak et al. (2001) showed a significant ∼4.5-fold increase in Penh following 48 h exposure to 100% O_2_ which did not correlate with a slight decrease in resistance measured in the same animals (Petak et al., 2001), in our study no significant change neither in EF_50_ nor in lung resistance was measured in the same animals after 48 h exposure to 100% O_2_ (see Figure 8; Glaab et al., 2005). Therefore, in contrast to the Penh results, head-out plethysmography has been proven to provide a reliable correlation between EF_50_ and pulmonary resistance.

**Table 3:** **Short comparison of Penh with tidal midexpiratory flow (EF_50_)**.

Penh	EF_50_
Conscious, freely moving animal	Conscious, restrained animal
For screening purposes, ca. up to 30–60 animals/day/technician	For screening purposes, ca. up to 32 animals/day/technician
No established physiological parameter	Physiological parameter (expiratory flow)
Dimensionless	mL/s
**Correlation with more direct measurements of pulmonary resistance (R_L_)**	
Sometimes fail to correlate^1^	Correlates with invasive measurement of R_L_ in several bronchoconstriction models^2^
Penh ↑ and VT ↑ during bronchoconstr. (!)^3^	EF_50_ ↓ and VT ↓ during bronchoconstriction^4^

*^1^Petak et al. (2001); DeLorme and Moss (2002); Lundblad et al. (2002); Flandre et al. (2003); Adler et al. (2004); Pauluhn (2004)*.

*^2^Neuhaus-Steinmetz et al. (2000); Glaab et al. (2001, 2002, 2005, 2006)*.

*^3^Hamelmann et al. (1997)*.

*^4^Lai and Chou (2000); Glaab et al. (2002, 2005)*.

*Reprinted with permission from Hoymann (2007), modified*.

Additionally, Legaspi et al. (2010) reported a concurrent validation of volume, rate, time, flow, and ratio variables in head-out plethysmography. They confirmed “the suitability of head-out plethysmography in rats for respiratory safety pharmacology as previously reported by Hoymann (2006)” and found flow derived parameters such as EF_50_ as “highly valuable complement for interpretation of respiratory response”. In an infection model, head-out plethysmography has been reported to be very useful for monitoring infection with *Pseudomonas aeruginosa* in mice showing a decrease in *V*_T_ and EF_50_ (Wölbeling et al., 2010). Recently, Nirogi et al. (2012) compared whole body and head-out plethysmography using respiratory stimulant and repressant in conscious rats and concluded that “ventilatory function can be accurately assessed using head-out plethysmography compared to whole body plethysmography”.

The ICH guideline S7A defines the respiratory system as a “vital organ system” that is considered one of the most critical ones, and as such should be assessed with the same scientific rigor as the other organ systems (i.e., CNS and cardiovascular; Nirogi et al., 2012). Therefore, the ability to accurately and reliably evaluate respiratory function in animals has become increasingly important (Renninger, 2006). Since the intent of safety pharmacology is to minimize the human risk on one hand but also to minimize the unnecessary removal of potentially useful drugs from the further development on the other hand, only such methods should be used which are proven to be reliable and are well validated for detecting adverse effects. Since it has been shown (see above) that Penh and the barometric plethysmography in some cases predicted false-adverse effects and is therefore and due to lack of a theoretical basis not really predictable for human risk, this leads to the conclusion that this method is not suitable for the safety pharmacology core battery. In contrast, the head-out plethysmography including the parameter EF_50_ has been proven to be a valid and reliable method (Vijayaraghavan et al., 1994; Glaab et al., 2002, 2005) which is easy to use, allows high throughput measurements, and was recommended to determine lung function non-invasively (Hantos and Brusasco, 2002; Glaab et al., 2005, 2007; Hoymann, 2006) which was recently confirmed by results of methodological comparison studies of Legaspi et al. (2010) and Nirogi et al. (2012). Therefore, head-out plethysmography is recommended to be used in the core battery of safety pharmacology testing (Renninger, 2006; Hoymann, 2007; Legaspi et al., 2010; Nirogi et al., 2012).

## Conclusion

The intent of safety pharmacology is to minimize the human risk on one hand but also to minimize the unnecessary removal of drugs from the development line on the other hand. Therefore only such methods should be used which are proven to be reliable and are well validated for detecting adverse effects on the respiratory system. The head-out plethysmography including EF_50_ measurement fulfill this requirement: it is proven as a valid and reliable method, is easy to use, allows measurements with a relatively high throughput, and is therefore recommended to be used in the core battery of safety pharmacology testing. It has also been shown to be successfully used in high throughput studies using, e.g., asthma models, in lung function measurements in infection models or as Alarie test on irritant potential in mice. In contrast, since it has been shown that the barometric plethysmography (Penh) in some cases predicted false-adverse effects and leads to misinterpretation, it is recommended not to use this technique without an independent assessment of airway resistance.

In safety pharmacology follow-up studies, repetitive invasive lung function testing in orotracheally intubated rodents is recommended since the most sensitive and specific parameters such as lung resistance and dynamic compliance are required. This technique allows also extremely controlled inhalation administration directly into the lungs – under exclusion of other pathways – or with high deposition doses. It has also been used extensively in pre-clinical studies using, e.g., asthma or infection models, e.g., to assess airway hyperresponsiveness.

## Conflict of Interest Statement

The author declares that the research was conducted in the absence of any commercial or financial relationships that could be construed as a potential conflict of interest.
